# Personal

**Published:** 1910-10

**Authors:** 


### PERSONAL

---

Dr. Charles N. Palmer, of Lockport, who sustained an automobile injury in the early summer at Cambria, is reported as entirely recovered. He is taking up his professional work again with his accustomed energy.

---

Dr. H. E. Hayd, of Buffalo, was elected president of the American Association of Obstetricians and Gynecologists at its twenty-third annual meeting recently held at Syracuse. Dr. Hayd for many years has been an efficient worker in the ranks of this famous association and has been deservedly honored by his elevation to the presidency.

---

Dr. Francis E. Fronczak, health commissioner of Buffalo, was elected Grand Medical Examiner of the Polish Union of America, at its annual meeting recently held at Niagara Falls. Dr. Fronczak was also awarded a prize of $250 in recognition of his services in behalf of the organization.

---

Dr. Burton T. Simpson, of Buffalo, passed the competitive examination—the only one to do so—for clinical pathologist at the State Cancer Laboratory in this city, the salary being $2,500 a year.

Dr. T. J. Sheehan, of Buffalo, recently sailed for Europe to be absent six weeks.

